# Differential infiltration of neutrophils in T1–T2 versus T3–T4 oral squamous cell carcinomas: a preliminary study

**DOI:** 10.1186/s13104-015-1541-x

**Published:** 2015-10-14

**Authors:** Patrícia Carlos Caldeira, Alexandre de Andrade Sousa, Maria Cássia Ferreira de Aguiar

**Affiliations:** Department of Oral Pathology and Surgery, School of Dentistry, Universidade Federal de Minas Gerais, Av. Antônio Carlos, 6627, Pampulha, CEP: 31.270-901 Belo Horizonte, MG Brazil; Department of Surgery of the School of Medicine and Head and Neck Surgery, Instituto Alfa de Gastroenterologia, Hospital das Clínicas, Universidade Federal de Minas Gerais, Belo Horizonte, MG Brazil

**Keywords:** Squamous cell carcinoma, Mouth neoplasms, Neutrophils, Immunohistochemistry

## Abstract

**Background:**

Recent studies have pointed towards a role of tumour-infiltrating neutrophils in cancer biology. Investigations on oral squamous cell carcinoma have indicated a possible association with clinical characteristics. This study aimed to evaluate neutrophil infiltration and the neutrophil/lymphocyte ratio in the central areas and invasive front of oral squamous cell carcinomas at different T stages, and their association with clinicopathological features and patient outcome.

**Methods:**

Clinical information was retrieved from the charts of patients who had undergone surgical treatment. Samples of the excised tumours were subjected to immunohistochemical analysis for CD66b and CD3. Semi-quantitative analysis was performed in the intratumoural region and in the invasive front. Appropriate statistical tests were used for evaluating the data, including Kaplan–Meier survival analysis and the log-rank test. A *p* value of less than 0.05 was considered significant.

**Results:**

T3–T4 tumours presented higher CD66b infiltration in the intratumoural region and higher CD66b/CD3 ratios in the invasive front than T1–T2 lesions (p < 0.05). There was a strong inverse correlation between CD66b and CD3 in the invasive front of T3–T4 tumours (r = −0.712, p < 0.05). Comparisons of CD66b and the CD66b/CD3 ratio according to N status, tumour location, recurrence, inflammation grade, and histological grade did not reach statistical significance. Survival analysis also did not show any significant differences.

**Conclusions:**

The present study showed different degrees of neutrophil infiltration between T1–T2 and T3–T4 oral cancers, with higher indexes in the advanced lesions. However, there was no association with clinicopathological features or with time to recurrence.

**Electronic supplementary material:**

The online version of this article (doi:10.1186/s13104-015-1541-x) contains supplementary material, which is available to authorized users.

## Background

Squamous cell carcinoma (SCC) is the most frequent cancer of the oral cavity, affecting mainly elderly men with a smoking habit. Despite therapeutic advances, the overall estimated 5-year survival is 56 % [1]. Currently, the TNM system is used as the prognostic indicator for SCC patients, but its performance is suboptimal in some cases [2].

Evidence points towards a role of tumour-associated leucocytes in the development and progression of malignant neoplasms, as these cells may exert stimulatory and inhibitory functions in the microtumour milieu [3–5]. Despite being previously neglected, neutrophils have now arisen as an important leucocyte in cancer biology, with pro- and anti-tumour functions [3, 5–12].

Tumour-associated neutrophils (TANs) may be detected in the intratumour, peritumour, or tumour-surrounding regions, and some studies have reported a clinical relevance of neutrophil infiltration in diverse cancers [12–17]. In a recently published review on this topic, Donskov et al. [18] reported the prognostic value of a high neutrophil count and neutrophil/lymphocyte ratio (NLR).

In head and neck carcinoma, T4 tumours presented higher neutrophil infiltration than T1 and T2 types [19]. Patients staged as III and IV with low amounts of TANs had a better 5-year survival than those with high neutrophil infiltration [19].

Some studies have exploited the participation and clinical relevance of TANs in oral SCC. A high NLR was associated with poor prognosis [12, 20, 21], poor tumour differentiation [20], and higher clinical stages [12, 22]. Moreover, a high neutrophil density was associated with lymph node metastasis and recurrence [12] and poor differentiation [21]. On the other hand, these studies reported a lack of association between TANs and age [12, 20, 21], tumour location [21], tumour size [12, 21], N stage [21], tumour grade [12], and clinical stage [20].

Considering the relevant role of neutrophils within the tumoural environment and the emerging results on TANs and oral SCC, we aimed to evaluate the neutrophil infiltration and NLR in oral SCCs at different T stages. Taking into account the intratumoural heterogeneity, neutrophils and lymphocytes were evaluated in the central areas and invasive front of the tumours. Additionally, we explored the possible association of these indexes with tumour grade, clinical features, and patient outcome.

## Methods

### Samples

The study was approved by the Committee of Ethics in Research of the Universidade Federal de Minas Gerais (28773114.7.0000.5149). Patients attending the Head and Neck Surgery Ambulatory Department gave written consent for the use of their registered data and tumour specimens in research developed at the Universidade Federal de Minas Gerais.

The files of the Head and Neck Surgery Department of the Hospital das Clínicas of the Universidade Federal de Minas Gerais were reviewed. Cases of patients who had undergone surgical treatment for oral SCC were selected, and clinical information was retrieved from the charts. Collected data included age, sex, smoking habit, alcohol consumption, lesion location, surgical treatment, radiotherapy, N stage, and follow-up.

The tumour specimens of these patients were retrieved from the files of the Laboratory of Pathological Anatomy of the same hospital. Paraffin blocks containing the invasive front region were used for tumour grading and immunohistochemical analysis.

### Histopathological grading of tumours

Slide samples stained with haematoxylin and eosin were independently reviewed by two oral pathologists (P.C.C. and M.C.F.A.), who graded the lesions according to the criteria established by Bryne et al. [23]. Discrepancies were resolved via discussion of the cases. At this point in the study, the pathologists were unaware of the T stage of the lesions.

### Inflammation grading

The amount of inflammatory infiltrate present in each slide (irrespective of cell type or immunostaining) was graded according to Bryne et al. [23] criteria as absent, weak, moderate, or intense.

### Immunohistochemistry

Tissues were subjected to immunohistochemical analysis for CD66b (1:600, clone GF10F5; BD Biosciences, EUA, code 555723) and CD3 (1:200, clone F7.2.38; Dako Cytomation, Denmark, code M7254). Sections were deparaffinised in xylol and hydrated in an ethanol solution. Antigen retrieval was performed with a TRIS–EDTA solution (pH 9.0) in a 96 °C water bath for 30 min. The hydrogen peroxide blocking, protein blocking, and detection steps were performed with ready-to-use solutions provided in the kit (Spring BioScience, SPB-999). The reaction was revealed with 3,3′-diaminobenzidine (Spring BioScience, code DAB-999), and haematoxylin was used for the counterstaining. Appropriate positive and negative controls were included.

### Evaluation of immunohistochemistry

One observer (P.C.C.) evaluated all cases using an optical microscope (Zeiss Axiostar Ser. 48824). A semi-quantitative analysis was done, based on the reports by Lundqvist et al. [24] and Shinriki et al. [21]. CD66b^+^ cells located inside blood vessels were not considered for quantification. Five high-power fields (400× magnification) were analysed separately in the invasive front and in the intratumoural region (Additional files 1, 2), and positivity was graded as 0 (no staining), 1 (1–25 % of positive cells), 2 (25–50 %), and 3 (>50 %). The mean value of the scores from the five fields was set as the CD66b and CD3 index of staining for each case. The CD66b/CD3 ratio was obtained by dividing the CD66b and CD3 indexes of each slide.

### Statistical analysis

Statistical tests were performed using SPSS^®^ version 19.0 for Windows and GraphPad Prism^®^ version 6.0 for Windows.

Firstly, the Shapiro–Wilk normality test was applied. Comparisons of clinical and microscopic features between the T1–T2 and T3–T4 groups were performed with the Pearson χ^2^ test.

The *t* test was used for comparisons of the CD66b and CD3 indexes between the T1–T2 and T3–T4 groups. The Spearman test was used to analyse the correlation between the CD66b and CD3 indexes.

Kruskal–Wallis and Mann–Whitney tests were used to compared the CD66b staining indexes and the CD66b/CD3 ratio between cases grouped according to N status (positive vs. negative), tumour location (tongue vs. floor of the mouth), recurrence (yes vs. no), inflammation grade [from Bryne classification: grade 1 (intense), 2 (moderate), 3 (weak)], and histological grade (from Bryne classification: I, II, III).

For the survival analysis, recurrence was the event of interest. Thus, the Kaplan–Meier method was used to evaluate patients grouped according to T stage (T1–T2 vs. T3–T4), CD66b index, and CD66b/CD3 ratio (the latter 2 being classified into low vs. high, according to median values). The log-rank test was used for comparisons.

For all the tests described above, a p value of less than 0.05 was considered significant.

## Results

Twenty-eight patients were included in the study. They were mainly males (n = 20, 71.4 %) above 50 years of age (n = 21, 75.0 %). Smoking habit (current or previous) was reported by most individuals (n = 25, 89.3 %), as well as alcohol consumption (n = 23, 82.1 %). Lesions were located in the tongue (n = 13, 46.4 %) or floor of the mouth (n = 15, 53.6 %). Lymph node involvement was observed in nine cases (32.1 %). With regard to treatment modality, tumour resection and unilateral neck dissection were performed in most cases (n = 15, 53.6 %), and adjuvant radiotherapy was used in 15 patients (53.6 %). The mean follow-up was 29 months (range 1–92 months) and 9 tumours recurred (32.1 %).

Most lesions were histologically classified as grade II (n = 21, 75.0 %). Inflammatory infiltrate was present in all cases and graded as intense in 10 cases (35.7 %), moderate in 12 (42.9 %), and weak in 6 (21.4 %).

Furthermore, cases were grouped according to T stages for comparisons. The detailed clinical and histopathological data are presented in Table 1. One group comprised 13 patients (46.4 %) with T1 and T2 tumours, whereas the other group was composed of 15 patients with T3 and T4 tumours (53.6 %). Surgical treatment, adjuvant radiotherapy, and lymph node involvement differed between the 2 groups (p < 0.05, Pearson χ^2^ test; Table 1). Patients with T1–T2 lesions were usually treated with tumour resection, unilateral neck dissection, and without adjuvant radiotherapy, whereas tumour resection, bilateral neck dissection, and radiotherapy were performed for T3–T4 lesions. Accordingly, lymph node involvement was rare in T1–T2 tumours, but not in T3–T4.

**Table 1:** Clinical and microscopic characteristics of the patients with oral squamous cell carcinoma

	T1–T2 (%)	T3–T4 (%)	Pearson χ^2^*
Age			0.535
<50 years	2 (15.4)	4 (26.7)	
≥50 years	10 (76.9)	11 (73.3)	
Data not available	1 (7.7)	–	
Sex			0.281
Male	8 (61.5)	12 (80.0)	
Female	5 (38.5)	3 (20.0)	
Smoking habit			0.292
Yes	4 (30.8)	9 (60.0)	
No	2 (15.4)	1 (6.7)	
Previous	7 (53.8)	5 (33.3)	
Alcohol consumption			0.796
Yes	5 (38.5)	4 (26.7)	
No	2 (15.4)	3 (20.0)	
Previous	6 (46.1)	8 (53.3)	
Lesion location			0.136
Tongue	8 (61.5)	5 (33.3)	
Floor of the mouth	5 (38.5)	10 (66.7)	
Surgical treatment			0.016**
Tumour resection	2 (15.4)	1 (6.7)	
Tumour resection and unilateral neck dissection	10 (76.9)	5 (33.3)	
Tumour resection and bilateral neck dissection	1 (7.7)	9 (60.0)	
Adjuvant radiotherapy			0.000**
Yes	1 (7.7)	14 (93.3)	
No	11 (84.6)	1 (6.7)	
Data not available	1 (7.7)	–	
Lymph node involvement			0.010**
Yes	1 (7.7)	8 (53.3)	
No	12 (92.3)	7 (46.7)	
Recurrence			0.149
Yes	5 (38.5)	4 (26.7)	
No	6 (46.1)	11 (73.3)	
Data not available	2 (15.4)	–	
Bryne classification			0.635
Grade I (4–8)	3 (23.1)	3 (20.0)	
Grade II (9–12)	10 (76.9)	11 (73.3)	
Grade III (13–16)	0	1 (6.7)	

* Calculated with valid cases only; ** statistically significant

**Fig. 1:**
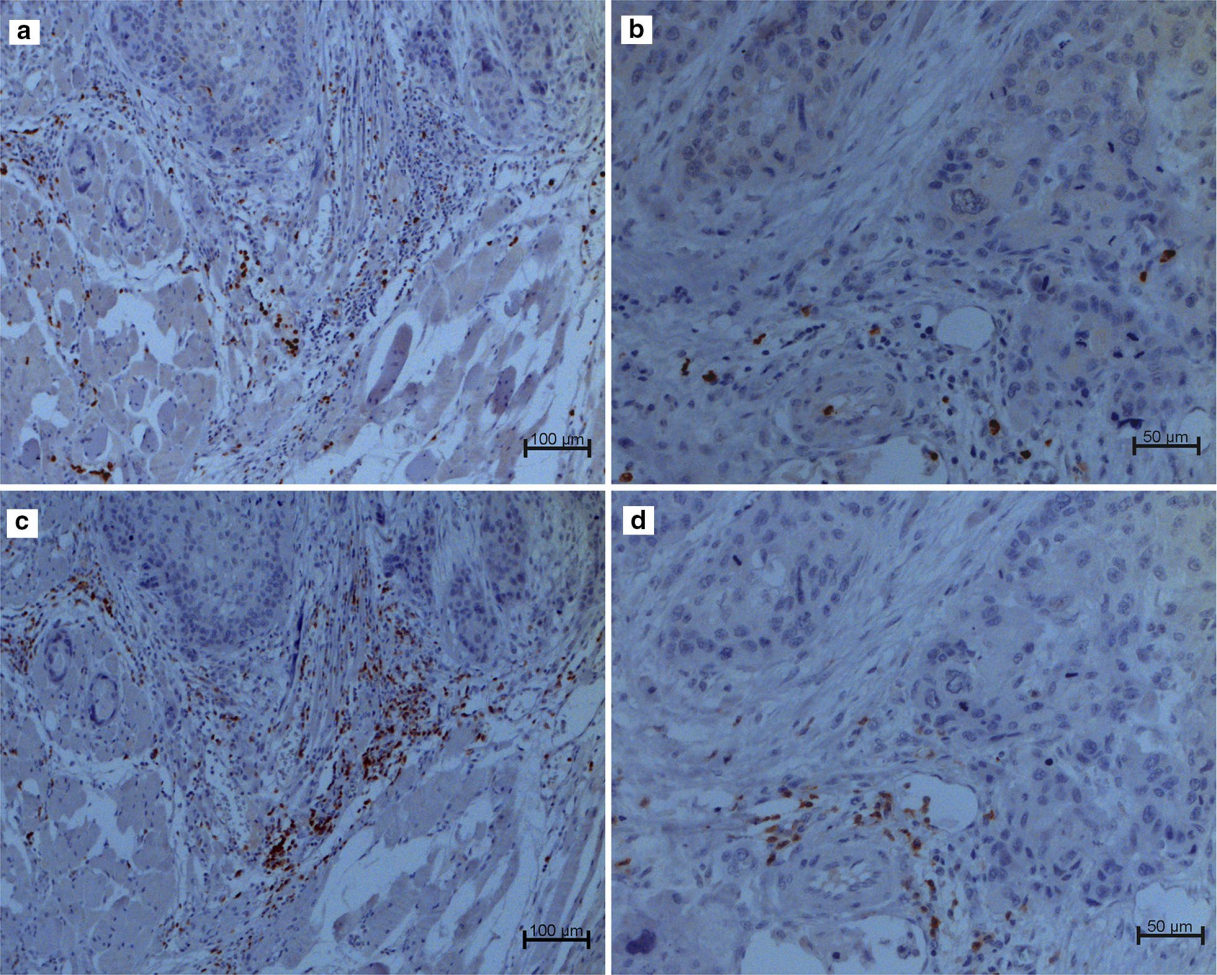
Tongue squamous cell carcinoma. Immunohistochemical detection of CD66b^+^ neutrophils in the invasive front **(a)** and intratumoural region **(b)** and CD3^+^ lymphocytes in the invasive front **(c)** and intratumoural region **(d)**. Original magnification: **a**, **c** = ×100; **b**, **d** = ×200

With regard to the immunostaining, CD66b^+^ cells were detected in all but 1 case (Additional file 1), whereas CD3^+^ lymphocytes were invariably present (Additional file 2). The immunostaining results can be observed in Fig. 1.

**Fig. 2:**
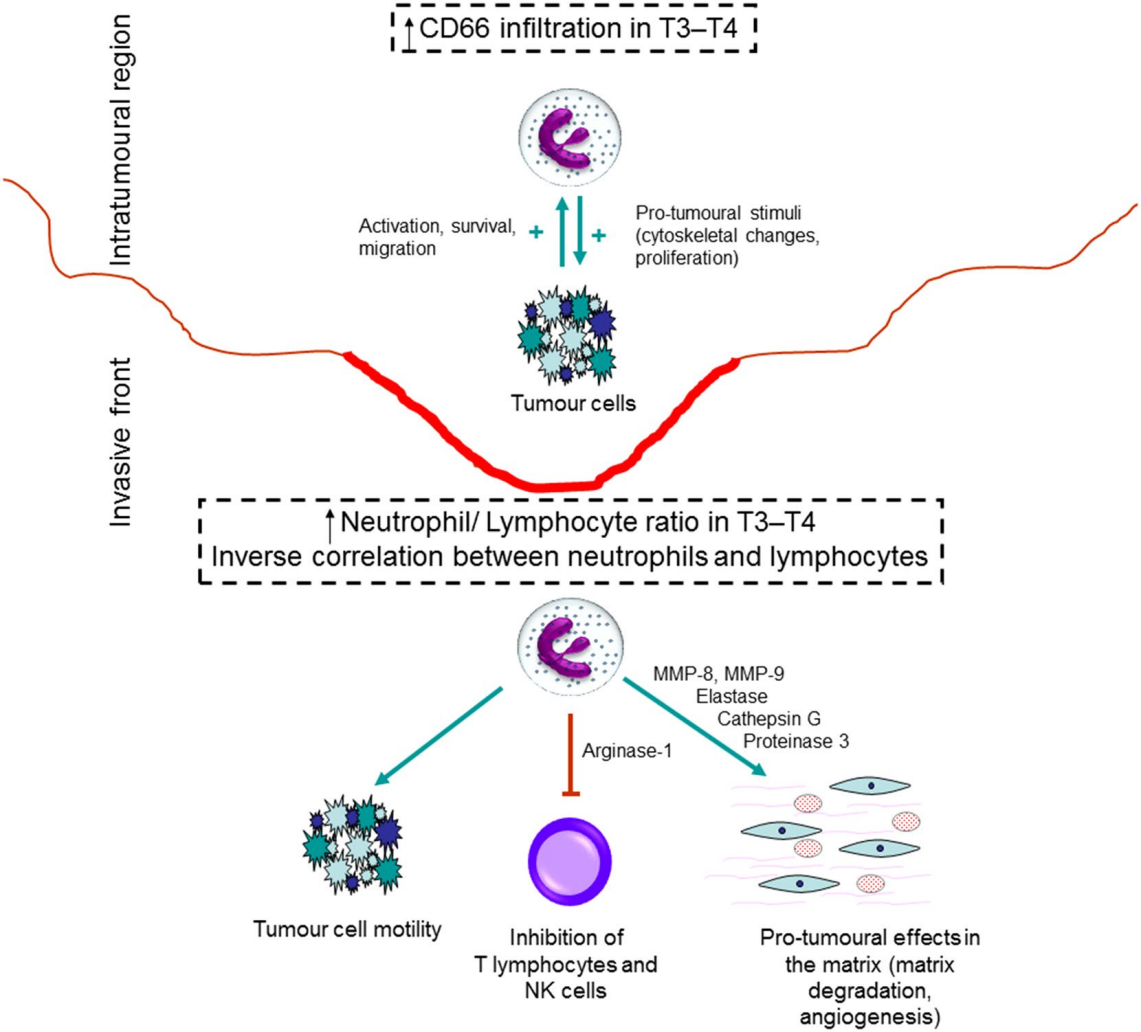
The differential infiltration of neutrophils in T3–T4 oral squamous cell carcinomas in the intratumoural region and invasive front found in the present study may be related to their diverse functions within the tumoural microenvironment, interacting with cancer cells, lymphocytes, and the extracellular matrix (see the “Discussion” section for further information)

Statistical comparisons of the immunostaining indexes showed that the T3–T4 tumours presented higher CD66b^+^ indexes in the intratumoural region and higher CD66b/CD3 ratios in the invasive front than did the T1–T2 lesions (p < 0.05, *t* test; Table 2; Fig. 2). Additionally, there was a strong and an inverse correlation between the CD66b and CD3 infiltration indexes in the invasive front of the T3–T4 tumours (r = −0.712, p < 0.05, Spearman test; Fig. 2).

**Table 2:** CD66b infiltration index and CD66b/CD3 ratio in T1–T2 versus T3–T4 tumours

	Invasive front	Intratumoural region
CD66b infiltration index		
T1–T2	0.86 ± 0.56	0.41 ± 0.38
T3–T4	1.33 ± 0.89	1.17 ± 1.03
p value	0.101	0.015*
CD66b/CD3 ratio		
T1–T2	0.36 ± 0.28	0.31 ± 0.27
T3–T4	0.85 ± 0.79	0.61 ± 0.57
p value	0.034*	0.084

Results are shown as mean values ± standard deviation; p values refer to the *t* test; * statistically significant

Comparisons of the CD66b infiltration index and CD66b/CD3 ratio according to N status, tumour location, recurrence, inflammation grade, and histological grade did not reach statistical significance (p > 0.05, Kruskal–Wallis and Mann–Whitney tests).

**Fig. 3:**
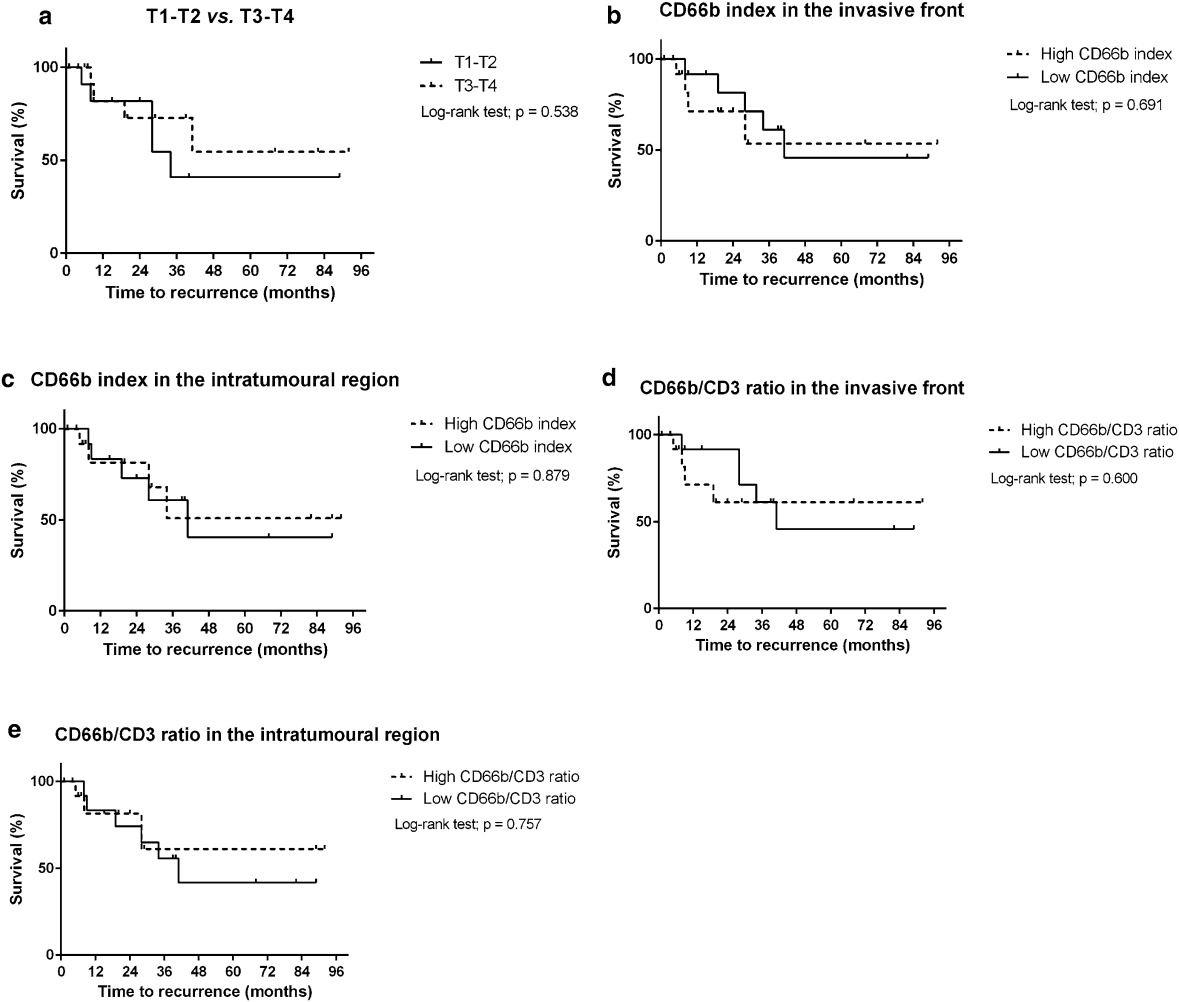
Survival curves (Kaplan–Meier method) of patients classified according to **a** T stage, **b** CD66b index in the invasive front, **c** CD66b index in the intratumoural region, **d** CD66b/CD3 ratio in the invasive front, and **e** CD66b/CD3 ratio in the intratumoural region. There was no difference in time to recurrence between the groups for any comparison (p > 0.05, log-rank test)

The time to recurrence was evaluated in the survival analysis, where comparisons were performed as follows (Table 3; Fig. 3): T1–T2 vs. T3–T4 lesions, high vs. low CD66b index in the invasive front; high vs. low CD66b index in the intratumoural region; high vs. low CD66b/CD3 ratio in the invasive front; and high vs. low CD66b/CD3 ratio in the intratumoural region. For all these comparisons, there were no statistically significant differences (p > 0.05, log-rank test; Table 3; Fig. 3).

**Table 3:** Survival analyses

	Number of cases	Months of follow-up (mean)	Number of events (recurrences)	Months until recurrence (mean)	p value*
T stage					0.538
T1–T2	13	22.0	5	20.6	
T3–T4	15	29.8	4	19.3	
CD66b index in invasive front					0.691
High	13	24.5	4	12.5	
Low	15	35.6	5	26.0	
CD66b index in intratumoural region					0.879
High	14	30.3	4	18.8	
Low	14	28.1	5	21.0	
CD66b/CD3 ratio in invasive front					0.600
High	14	22.2	4	10.3	
Low	14	36.2	5	27.8	
CD66b/CD3 ratio in intratumoural region					0.757
High	14	24.8	3	13.7	
Low	14	33.6	6	23.2	

* Survival analysis was performed using the Kaplan–Meier method and log-rank test

## Discussion

In the present study, advanced oral SCCs presented a higher infiltration of TANs within the intratumoural region than did initial lesions. Similarly, Trellakis et al. [19] reported that advanced head and neck SCCs were infiltrated by neutrophils, and T4 lesions showed higher infiltration than T1–T3 tumours. Wang et al. [12] also identified intratumoural CD15^+^ neutrophils in tongue SCCs.

The presence of neutrophils within the intratumoural area may be related to their functions within cancer biology (Fig. 2) [25]. The cross-talk between neutrophils and cancer cells may be related to cancer cell motility [26], migratory activity [27], cytoskeletal changes [28], and tumour cell proliferation [28, 29]. In turn, cancer cells may have feedback effects on neutrophils, increasing their migration and survival, or leading to a different activation status [19, 25, 26, 28, 30]. Further investigation on the molecules and pathways involved in the cross-talk between cancer cells and neutrophils may clarify the participation of TANs in oral SCC pathogenesis.

Recent studies have encompassed the role of myeloid-derived suppressor cells (MDSCs) in cancer [31–33]. MDSCs comprise a heterogeneous cell population with immunosuppressor function, for which there is no specific marker (or even a defined and exclusive panel of markers) [31–33]. One subset of MDSCs, the granulocytic MDSCs, shares overlapping features with neutrophils with regard to cell morphology (polymorphonuclear granulocytic cells), phenotype (both are CD15 and CD66b positive), and function (e.g. arginase-1 and myeloperoxidase production) [33]. Although the immunosuppressive activity is regarded as a hallmark of granulocytic MDSCs, neutrophils may also exert immunosuppressive activity if activated [31, 33]. Therefore, distinguishing between neutrophils and granulocytic MDSCs may be broadly difficult [33]. Accordingly, some cells named “neutrophils” in the present study could reasonably represent MDSCs instead. In any case, as stated by Brandau et al. [33], tumour-associated neutrophils and granulocytic MDSCs guard close resemblances to each other and may ‘represent similar functional states of cells originating from the same cell type’.

Tumours should be regarded as heterogeneous masses [34], and the invasive front is considered the most aggressive area [35]. In the present study, there was a higher NLR in the invasive front of T3–T4 tumours, and an inverse correlation between neutrophils and T lymphocytes was detected (Fig. 2). Likewise, Tsai et al. [22] showed a decrease in lymphocytes and an increase in neutrophil counts with the advancement of clinical stage of oral SCCs. Neutrophils may influence the recruitment and function of CD8^+^ and CD4^+^ T lymphocytes, both important cells in the cancer microenvironment. Besides this, T lymphocytes can modulate neutrophil function [28, 36–41]. Neutrophils can also contribute to matrix re-modelling and angiogenesis in the invasive front, favouring cancer spread [13, 25, 42–44]. Interestingly, it has been shown that neutrophils can be recruited to niches of distant metastasis and may participate in the metastatic spread of cancer [45].

In the present study, we failed to find any association between neutrophils and clinicopathological features, including in the survival analysis. Previous studies reported associations with poor prognosis [12, 20, 21], poor tumour differentiation [21], higher clinical stages [12, 22], lymph node metastasis, and recurrence [12]. However, these observations are not unanimous in the literature [12, 20, 21]. Concerning these discrepancies, some points merit a few comments. Firstly, certain studies employed tissue microarrays, in which a limited area of the lesion was evaluated. In the present study, however, we evaluated the tumour as a whole. Importantly, we report differences in results between distinct tumour areas. Secondly, as a preliminary study, the limited number of cases included herein may have interfered with the results and statistical analysis, and further investigations are encouraged. Thirdly, the non-standardisation of immunohistochemical evaluation methods continues to pose challenges when comparing study results. Although quantitative, semi-quantitative, and computer-based analyses are suitable, all of them clearly have advantages and limitations.

## Conclusion

The present study showed different degrees of neutrophil infiltration between T1–T2 and T3–T4 oral SCCs, with higher indexes in the advanced lesions. There was no association with clinicopathological features or with time to recurrence. Knowledge on the clinical and pathological importance of TANs, especially in oral SCC, is starting to be built. Results reported to date are exciting and encourage further studies to explore the neutrophil-derived molecules present in tumours.

## Additional files

10.1186/s13104-015-1541-x Detailed scores of immunohistochemical evaluation of CD66b. This table reports the scores obtained for each slide field in all samples for the immunohistochemical evaluation of CD66b.
10.1186/s13104-015-1541-x Detailed scores of immunohistochemical evaluation of CD3. This table reports the scores obtained for each slide field in all samples for the immunohistochemical evaluation of CD3.

## Abbreviations

SCC: squamous cell carcinoma
TANs: tumour-associated neutrophils
NLR: neutrophil/lymphocyte ratio
MDSCs: myeloid-derived suppressor cells

## References

[1] Bagan J, Sarrion G, Jimenez Y (2010). Oral cancer: clinical features. Oral Oncol.

[2] Ebrahimi A, Gil Z, Amit M, Yen TC, Liao CT, Chaturvedi P (2014). Primary tumor staging for oral cancer and a proposed modification incorporating depth of invasion: an international multicenter retrospective study. JAMA Otolaryngol Head Neck Surg.

[3] Piccard H, Muschel RJ, Opdenakker G (2012). On the dual roles and polarized phenotypes of neutrophils in tumor development and progression. Crit Rev Oncol Hematol.

[4] Zhou C, Liu J, Tang Y, Liang X (2012). Inflammation linking EMT and cancer stem cells. Oral Oncol.

[5] Tecchio C, Scapini P, Pizzolo G, Cassatella MA (2013). On the cytokines produced by human neutrophils in tumors. Semin Cancer Biol.

[6] Fridlender ZG, Sun J, Kim S, Kapoor V, Cheng G, Ling L (2009). Polarization of tumor-associated neutrophil phenotype by TGF-beta: “N1” versus “N2” TAN. Cancer Cell.

[7] Houghton AM (2010). The paradox of tumor-associated neutrophils: fueling tumor growth with cytotoxic substances. Cell Cycle.

[8] Gregory AD, Houghton AM (2011). Tumor-associated neutrophils: new targets for cancer therapy. Cancer Res.

[9] Fridlender ZG, Albelda SM (2012). Tumor-associated neutrophils: friend or foe?. Carcinogenesis.

[10] Brandau S (2013). The dichotomy of neutrophil granulocytes in cancer. Semin Cancer Biol.

[11] Galdiero MR, Bonavita E, Barajon I, Garlanda C, Mantovani A, Jaillon S (2013). Tumor associated macrophages and neutrophils in cancer. Immunobiology.

[12] Wang N, Feng Y, Wang Q, Liu S, Xiang L, Sun M (2014). Neutrophils infiltration in the tongue squamous cell carcinoma and its correlation with CEACAM1 expression on tumor cells. PLoS One.

[13] Li YW, Qiu SJ, Fan J, Zhou J, Gao Q, Xiao YS (2011). Intratumoral neutrophils: a poor prognostic factor for hepatocellular carcinoma following resection. J Hepatol.

[14] Gu FM, Gao Q, Shi GM, Zhang X, Wang J, Jiang JH (2012). Intratumoral IL-17^+^ cells and neutrophils show strong prognostic significance in intrahepatic cholangiocarcinoma. Ann Surg Oncol.

[15] Rao HL, Chen JW, Li M, Xiao YB, Fu J, Zeng YX (2012). Increased intratumoral neutrophil in colorectal carcinomas correlates closely with malignant phenotype and predicts patients’ adverse prognosis. PLoS One.

[16] Zhao JJ, Pan K, Wang W, Chen JG, Wu YH, Lv L (2012). The prognostic value of tumor-infiltrating neutrophils in gastric adenocarcinoma after resection. PLoS One.

[17] Ilie M, Hofman V, Ortholan C, Bonnetaud C, Coëlle C, Mouroux J (2012). Predictive clinical outcome of the intratumoral CD66b-positive neutrophil-to-CD8-positive T-cell ratio in patients with resectable nonsmall cell lung cancer. Cancer.

[18] Donskov F (2013). Immunomonitoring and prognostic relevance of neutrophils in clinical trials. Semin Cancer Biol.

[19] Trellakis S, Bruderek K, Dumitru CA, Gholaman H, Gu X, Bankfalvi A (2011). Polymorphonuclear granulocytes in human head and neck cancer: enhanced inflammatory activity, modulation by cancer cells and expansion in advanced disease. Int J Cancer.

[20] Perisanidis C, Kornek G, Pöschl PW, Holzinger D, Pirklbauer K, Schopper C (2013). High neutrophil-to-lymphocyte ratio is an independent marker of poor disease-specific survival in patients with oral cancer. Med Oncol.

[21] Shinriki S, Jono H, Ueda M, Obayashi K, Nakamura T, Ota K (2014). Stromal expression of neutrophil gelatinase-associated lipocalin correlates with poor differentiation and adverse prognosis in oral squamous cell carcinoma. Histopathology.

[22] Tsai YD, Wang CP, Chen CY, Lin LW, Hwang TZ, Lu LF (2014). Pretreatment circulating monocyte count associated with poor prognosis in patients with oral cavity cancer. Head Neck.

[23] Bryne M, Koppang HS, Lilleng R, Kjaerheim A (1992). Malignancy grading of the deep invasive margins of oral squamous cell carcinomas has high prognostic value. J Pathol.

[24] Lundqvist L, Stenlund H, Laurell G, Nylander K (2012). The importance of stromal inflammation in squamous cell carcinoma of the tongue. J Oral Pathol Med.

[25] Magalhaes MA, Glogauer JE, Glogauer M (2014). Neutrophils and oral squamous cell carcinoma: lessons learned and future directions. J Leukoc Biol.

[26] Wu Y, Zhao Q, Peng C, Sun L, Li XF, Kuang DM (2011). Neutrophils promote motility of cancer cells via a hyaluronan-mediated TLR4/PI3K activation loop. J Pathol.

[27] Strell C, Lang K, Niggemann B, Zaenker KS, Entschladen F (2010). Neutrophil granulocytes promote the migratory activity of MDA-MB-468 human breast carcinoma cells via ICAM-1. Exp Cell Res.

[28] Dumitru CA, Lang S, Brandau S (2013). Modulation of neutrophil granulocytes in the tumor microenvironment: mechanisms and consequences for tumor progression. Semin Cancer Biol.

[29] Souto JC, Vila L, Brú A (2011). Polymorphonuclear neutrophils and cancer: intense and sustained neutrophilia as a treatment against solid tumors. Med Res Rev.

[30] Wang J, Arase H (2014). Regulation of immune responses by neutrophils. Ann N Y Acad Sci.

[31] Greten TF, Manns MP, Korangy F (2011). Myeloid derived suppressor cells in human diseases. Int Immunopharmacol.

[32] Gabrilovich DI, Ostrand-Rosenberg S, Bronte V (2012). Coordinated regulation of myeloid cells by tumours. Nat Rev Immunol.

[33] Brandau S, Moses K, Lang S (2013). The kinship of neutrophils and granulocytic myeloid-derived suppressor cells in cancer: cousins, siblings or twins?. Semin Cancer Biol.

[34] Wang X, Fan M, Chen X, Wang S, Alsharif MJ, Wang L (2006). Intratumor genomic heterogeneity correlates with histological grade of advanced oral squamous cell carcinoma. Oral Oncol.

[35] Bryne M (1998). Is the invasive front of an oral carcinoma the most important area for prognostication?. Oral Dis.

[36] de Oca RM, Buendía AJ, Del Río L, Sánchez J, Salinas J, Navarro JA (2000). Polymorphonuclear neutrophils are necessary for the recruitment of CD8(+) T cells in the liver in a pregnant mouse model of *Chlamydophila abortus* (*Chlamydia psittaci* serotype 1) infection. Infect Immun.

[37] Tvinnereim AR, Hamilton SE, Harty JT (2004). Neutrophil involvement in cross-priming CD8+ T cell responses to bacterial antigens. J Immunol.

[38] Kousis PC, Henderson BW, Maier PG, Gollnick SO (2007). Photodynamic therapy enhancement of antitumor immunity is regulated by neutrophils. Cancer Res.

[39] Iking-Konert C, Vogl T, Prior B, Wagner C, Sander O, Bleck E (2008). T lymphocytes in patients with primary vasculitis: expansion of CD8+ T cells with the propensity to activate polymorphonuclear neutrophils. Rheumatology (Oxford).

[40] Li KJ, Lu MC, Hsieh SC, Wu CH, Yu HS, Tsai CY (2006). Release of surface-expressed lactoferrin from polymorphonuclear neutrophils after contact with CD4+ T cells and its modulation on Th1/Th2 cytokine production. J Leukoc Biol.

[41] Lewkowicz P, Lewkowicz N, Sasiak A, Tchórzewski H (2006). Lipopolysaccharide-activated CD4+ CD25+ T regulatory cells inhibit neutrophil function and promote their apoptosis and death. J Immunol.

[42] Pekarek LA, Starr BA, Toledano AY, Schreiber H (1995). Inhibition of tumor growth by elimination of granulocytes. J Exp Med.

[43] Jablonska J, Leschner S, Westphal K, Lienenklaus S, Weiss S (2010). Neutrophils responsive to endogenous IFN-beta regulate tumor angiogenesis and growth in a mouse tumor model. J Clin Invest.

[44] Kuang DM, Zhao Q, Wu Y, Peng C, Wang J, Xu Z (2011). Peritumoral neutrophils link inflammatory response to disease progression by fostering angiogenesis in hepatocellular carcinoma. J Hepatol.

[45] Spicer JD, McDonald B, Cools-Lartigue JJ, Chow SC, Giannias B, Kubes P (2012). Neutrophils promote liver metastasis via Mac-1-mediated interactions with circulating tumor cells. Cancer Res.

