# Supplementary material for: Differential infiltration of neutrophils in T1–T2 versus T3–T4 oral squamous cell carcinomas: a preliminary study
Source: BMC Res Notes. 2015 Oct 14;8:569. doi: 10.1186/s13104-015-1541-x (PMC4607015; doi:10.1186/s13104-015-1541-x)
Supplement: Supplementary file 2 — 10.1186/s13104-015-1541-x Detailed scores of immunohistochemical evaluation of CD3. This table reports the scores obtained for each slide field in all samples for the immunohistochemical evaluation of CD3. [file 13104_2015_1541_MOESM2_ESM.docx]

**Additional file 2 – Detailed scores of immunohistochemical evaluation of CD3.**

| Case | Region | Scores | | | | | |
| --- | --- | --- | --- | --- | --- | --- | --- |
|  |  | Field 1 | Field 2 | Field 3 | Field 4 | Field 5 | Mean |
| 1 | Invasive Front | 1 | 1 | 2 | 3 | 1 | 1.6 |
|  | Intratumoural | 2 | 1 | 2 | 2 | 1 | 1.6 |
| 2 | Invasive Front | 3 | 2 | 3 | 2 | 3 | 2.6 |
|  | Intratumoural | 3 | 3 | 2 | 2 | 1 | 2.2 |
| 3 | Invasive Front | 1 | 1 | 1 | 2 | 3 | 1.6 |
|  | Intratumoural | 2 | 3 | 3 | 2 | 2 | 2.4 |
| 4 | Invasive Front | 1 | 1 | 1 | 1 | 2 | 1.2 |
|  | Intratumoural | 2 | 2 | 2 | 2 | 1 | 1.8 |
| 5 | Invasive Front | 2 | 2 | 2 | 2 | 1 | 1.8 |
|  | Intratumoural | 2 | 2 | 2 | 2 | 0 | 1.6 |
| 6 | Invasive Front | 1 | 2 | 1 | 1 | 1 | 1.2 |
|  | Intratumoural | 2 | 2 | 3 | 1 | 1 | 1.8 |
| 7 | Invasive Front | 3 | 1 | 2 | 2 | 1 | 1.8 |
|  | Intratumoural | 3 | 2 | 2 | 1 | 2 | 2.0 |
| 8 | Invasive Front | 2 | 3 | 3 | 3 | 3 | 2.8 |
|  | Intratumoural | 3 | 2 | 3 | 2 | 2 | 2.4 |
| 9 | Invasive Front | 2 | 1 | 0 | 0 | 2 | 1.0 |
|  | Intratumoural | 3 | 2 | 2 | 2 | 2 | 2.2 |
| 10 | Invasive Front | 2 | 2 | 3 | 3 | 2 | 2.4 |
|  | Intratumoural | 3 | 2 | 3 | 1 | 3 | 2.4 |
| 11 | Invasive Front | 3 | 3 | 3 | 3 | 2 | 2.8 |
|  | Intratumoural | 2 | 2 | 2 | 2 | 2 | 2.0 |
| 12 | Invasive Front | 3 | 3 | 3 | 3 | 3 | 3.0 |
|  | Intratumoural | 3 | 3 | 3 | 3 | 3 | 3.0 |
| 13 | Invasive Front | 3 | 2 | 1 | 2 | 1 | 1.8 |
|  | Intratumoural | 2 | 1 | 1 | 1 | 1 | 1.2 |
| 14 | Invasive Front | 3 | 3 | 2 | 2 | 3 | 2.6 |
|  | Intratumoural | 3 | 3 | 3 | 3 | 3 | 3.0 |
| 15 | Invasive Front | 3 | 3 | 3 | 3 | 2 | 2.2 |
|  | Intratumoural | 3 | 2 | 3 | 3 | 2 | 2.6 |
| 16 | Invasive Front | 3 | 3 | 3 | 3 | 3 | 3.0 |
|  | Intratumoural | 3 | 2 | 3 | 2 | 2 | 2.4 |
| 17 | Invasive Front | 3 | 3 | 2 | 3 | 2 | 2.6 |
|  | Intratumoural | 2 | 0 | 0 | 0 | 0 | 0.4 |
| 18 | Invasive Front | 3 | 3 | 2 | 3 | 2 | 2.6 |
|  | Intratumoural | 3 | 2 | 3 | 2 | 3 | 2.6 |
| 19 | Invasive Front | 3 | 3 | 2 | 3 | 3 | 2.8 |
|  | Intratumoural | 1 | 1 | 2 | 1 | 0 | 1.0 |
| 20 | Invasive Front | 3 | 3 | 3 | 3 | 3 | 3.0 |
|  | Intratumoural | 2 | 1 | 1 | 2 | 1 | 1.4 |
| 21 | Invasive Front | 3 | 3 | 3 | 3 | 3 | 3.0 |
|  | Intratumoural | 1 | 1 | 0 | 1 | 1 | 0.8 |
| 22 | Invasive Front | 3 | 3 | 2 | 2 | 2 | 2.4 |
|  | Intratumoural | 2 | 2 | 2 | 2 | 1 | 1.8 |
| 23 | Invasive Front | 3 | 3 | 3 | 3 | 3 | 3.0 |
|  | Intratumoural | 2 | 1 | 1 | 2 | 2 | 1.6 |
| 24 | Invasive Front | 3 | 3 | 3 | 3 | 2 | 2.8 |
|  | Intratumoural | 2 | 0 | 0 | 0 | 0 | 0.4 |
| 25 | Invasive Front | 2 | 2 | 3 | 3 | 3 | 2.6 |
|  | Intratumoural | 2 | 3 | 2 | 2 | 1 | 2.0 |
| 26 | Invasive Front | 0 | 1 | 2 | 1 | 1 | 1.0 |
|  | Intratumoural | 2 | 1 | 0 | 1 | - | 1.0 |
| 27 | Invasive Front | 3 | 3 | 3 | 3 | 3 | 3.0 |
|  | Intratumoural | 2 | 3 | 3 | - | - | 1.6 |
| 28 | Invasive Front | 3 | 2 | 3 | 3 | 3 | 2.8 |
|  | Intratumoural | 2 | 3 | 3 | 3 | 3 | 2.8 |
